# Flow-Adjusted Trabeculectomy

**DOI:** 10.3390/jcm13216609

**Published:** 2024-11-04

**Authors:** Assaf Kratz, Ivan Goldberg, Tal Koren, Aviel Hadad, Boris Knyazer, Ridia Lim

**Affiliations:** 1 Department of Ophthalmology, Soroka University Medical Center, Faculty of Health Sciences, Ben-Gurion University of the Negev, P.O. Box 151, Beer-Sheva 8400101, Israel; 2 Glaucoma Unit, Sydney Eye Hospital, GPO Box 1614, Sydney, NSW 2001, Australia; 3 Discipline of Ophthalmology, Sydney University, Sydney, NSW 2745, Australia; 4 Eye Associates, Sydney, NSW 2000, Australia; 5 Save Sight Institute, School of Medicine, The University of Sydney, Camperdown, NSW 2050, Australia

**Keywords:** trabeculectomy, intra-operative flow adjustment

## Abstract

**Background/Objectives:** As one of the most efficacious glaucoma surgical techniques, trabeculectomy is considered by many surgeons to be the “gold standard” intra-ocular pressure (IOP)-reducing intervention. The purpose of this study is to present our intra-operative flow-adjusted surgical method, which aims to provide safety and efficacy more simply than previous methods. **Methods:** Retrospectively, we evaluated outcomes for trabeculectomy or phacotrabeculectomy in surgery-naïve eyes over three years for patients with glaucoma not associated with other ocular co-morbidities. We defined complete success as an IOP between 5 and 18 mmHg plus at least a 20% reduction from baseline, without concomitant medications. Relative success was the same result, with glaucoma medication(s). Failure was regarded as an IOP less than 5 or higher than 18 mmHg, or by the need for a subsequent glaucoma operation. **Results:** We assessed the results from 186 eyes of 186 patients. After exclusion, a group of 45 trabeculectomies and 35 phacotrabeculectomies were analyzed. In eyes undergoing a trabeculectomy, over a mean follow-up of 16.0 months, IOP fell from 28.1 ± 8.0 mmHg with 3.6 ± 1.1 medications to 9.7 ± 3.6 mmHg (66% reduction) with 0.4 ± 1.0 medications (each *p* < 0.00001). The success rate was 88.9% (75.6% complete success). In eyes undergoing a phacotrabeculectomy, over a mean of 19.1 months, IOP fell from 26.1 ± 10.2 mmHg with 3.5 ± 1.3 medications to 10.0 ± 3.6 mmHg (62% reduction) on 0.9 ± 1.4 medications (each *p* < 0.00001). The success rate was 91.4% (57.1% complete success). Complication rates were low, with no major complications in either group. **Conclusion:** To lower IOP, our intra-operative flow-adjusted trabeculectomy and phacotrabeculectomy techniques appear to be safe and effective.

## 1. Introduction

Despite numerous advancements in glaucoma surgeries over the years, including the development of shunts, valves, angle-based procedures, and laser techniques, trabeculectomy, introduced by Cairns in 1968 [1], remains one of the most enduring and effective techniques for reducing intra-ocular pressure (IOP) in patients with glaucoma and ocular hypertension (OHT). The primary goals of this procedure are to maintain its efficacy in lowering IOP while ensuring the safety of the patient, both of which are crucial concerns for glaucoma surgeons and their patients alike. Over the decades, various modifications and enhancements have been made to the original technique to improve outcomes.

One notable advancement is the “Moorfields Safer Surgery System” developed by Khaw and colleagues [2,3]. This method has been instrumental in refining the trabeculectomy procedure by emphasizing the comprehensive use of anti-metabolites, such as mitomycin-C, in the sub-conjunctival space to reduce scar formation and enhance the longevity of the filtration bleb. Additionally, the method incorporates the use of an anterior chamber maintainer (ACM) to stabilize the eye during surgery. It also advocates for the use of four adjustable and two releasable sutures for the closure of the scleral flap, allowing for post-operative adjustment of IOP. Furthermore, specific suturing techniques are employed to ensure a water-tight closure of the conjunctiva, which is critical for preventing complications such as leaks or infections.

While the Moorfields technique has proven to be highly effective, it is not without its challenges. The procedure can be time-consuming due to the complexity of the scleral flap and conjunctival closure techniques. Additionally, the titration of IOP post-operatively relies heavily on the adjustment of the sutures or their removal, which must be done within a limited time window for optimal effectiveness. This can present a challenge in achieving the desired IOP control, as there is only a short period during which these modifications can be made.

Recognizing these challenges, we have evolved our surgical technique by integrating aspects of the Moorfields method with other innovative ideas. Our aim has been to increase the predictability, safety, and efficacy of the trabeculectomy procedure. In our approach, we focus on refining the surgical steps to reduce operative time while maintaining the precision and effectiveness of the surgery. By doing so, we strive to enhance the outcomes for our patients, ensuring that the procedure not only lowers IOP effectively but also minimizes the risks of complications.

In this report, we describe our modified trabeculectomy technique, outlining the specific steps and innovations we have introduced. Briefly, we utilize only two fixed sutures instead of the traditional combination of four adjustable and two releasable sutures. This simplification is made possible by our dynamic use of the ACM, which not only stabilizes the anterior chamber but also facilitates the safe execution of subsequent surgical steps and ensures adequate and secure filtration.

We also provide an analysis of our clinical results, highlighting the improvements in patient outcomes compared to traditional methods. Our goal is to contribute to the ongoing evolution of trabeculectomy, offering a technique that is both safer and more reliable for glaucoma patients who require surgical intervention to manage their condition.

## 2. Methods

### 2.1. Study Population

At the Soroka University Medical Center, Beer Sheva, Israel, we assessed results over a three-year period of either trabeculectomy or phacotrabeculectomy performed for patients with medically uncontrolled advanced glaucoma with or without significant cataract. Combined surgery was considered in cases with clinically significant cataract, or preemptively if significant cataract was likely in the short term and subsequent cataract surgery could jeopardize the functionality of an existing bleb. Ours is a tertiary referral center serving about one million people, composed of both Arabic and mixed-ethnic origin Jewish populations. We reviewed the medical records retrospectively. Our study was approved by the Institutional Review Board (IRB) (approval number: 0176-19-SOR) and adhered to the tenets of the Declaration of Helsinki. The IRB granted a full waiver of the requirement for an informed consent form for this study.

Subjects included individuals >18 years of age with medically uncontrolled primary open-angle glaucoma (POAG), exfoliative (XFG) glaucoma, or pigmentary glaucoma, with no other ocular pathology. Exclusion criteria were patients with any other form of glaucoma, or corneal or retinal co-morbidities. A power analysis determined that a minimum sample size of 31 eyes was required to detect a change of 0.5 ± 1 mmHg, with an alpha error of 0.05 and a power of 80%. We targeted a larger sample size to enhance the statistical power of the study, thereby improving the accuracy and reliability of our findings

For each patient, we collected demographic data, pre- and post-operative IOP, pre- and post-operative visual acuity, visual field mean deviation, duration of glaucoma diagnosis, follow-up period, and number of IOP-lowering medications. Post-operative procedures recorded included needlings and/or anti-metabolite injections. 

### 2.2. Surgical Procedures

All surgeries were performed by a single, fellowship-trained glaucoma specialist (AK) in the usual antiseptic manner, under peri- or retro-bulbar anesthesia. Key stages of the technique were: (a) corneal traction with a round-body 6/0 Vicryl suture; (b) sub-conjunctival injection of mixed bupivacaine/adrenaline solution; (c) deep posterior peritomy (i.e., fornix-based conjunctival flap), diathermy; (d) half-thickness, 4.0 mm × 3.5 mm rectangular scleral flap to 0.5 mm from the limbus; (e) application for 2.5 min of four small sponges soaked with 0.04% mitomycin C solution (MMC) deep into the conjunctival pouch: three far posterior into the conjunctival pouch, with the fourth sponge placed directly over the scleral flap. To facilitate removal, the sponges were pre-tied with 4/0 silk thread. During MMC application, the limbus area was protected from MMC solution and to minimize conjunctival edge contact with MMC, the conjunctiva was lifted upwards to create a “tent”; (f) thorough saline irrigation to remove all MMC; (g) anterior dissection to complete the scleral flap; (h) temporal insertion of a 20-gauge Blumenthal anterior chamber maintainer (ACM) as far as possible from the peritomy. The ACM bottle was adjusted ~50 cm above the patient’s eye; (i) insertion of two oblique 10/0 nylon sutures, one on each corner of the scleral flap, left untied; (j) lowering the ACM bottle to ~30 cm above the patient’s eye; (k) entering the anterior chamber under the scleral flap; (l) creating an ~0.5 mm punch sclerostomy; (m) performing an iridectomy limited to the iris tissue that is pushed against the sclerostomy; (n) first closure of the nylon sutures. (o) lowering the ACM to ~10 cm above the patient’s eye; (p) check for posterior filtration under the scleral flap, adjusting the sutures’ tension to allow minimal posterior oozing, to eliminate lateral oozing with lateral flap 10/0 nylon sutures as needed; (q) conjunctival closure with two 9/0 Vicryl sutures on a spatula needle, on each side of the peritomy. Check for water-tight closure; (r) remove the ACM and hydrate the paracentesis; (s) instill 3rd-or 4th-generation quinolone antibiotics, povidone iodine and atropine drops; (t) sub-conjunctival steroid injection; (u) eye pad with antibiotic ointment and eye shield. 

With phacotrabeculectomy surgery, after stage f above, a separate, 2.6 mm clear cornea incision is made, as far as possible from the peritomy; phacoemulsification is performed as usual. One 10/0 nylon suture seals the corneal incision, and carbachol intra-ocular solution is injected into the anterior chamber. The trabeculectomy then proceeds as described above. 

Adjustments in ACM height were made with the assistance of a non-sterile nurse, using the patient’s eye as a reference point. During the preparation of the two nylon sutures and the creation of the scleral punch, when maintaining a firm globe is beneficial, the ACM was positioned approximately 50 cm above the patient’s eye. To prevent excessive iris tissue prolapse and to achieve a well-shaped iridectomy, the ACM was lowered to about 30 cm above the eye before this stage. Finally, for fine titration of flow, the ACM was further reduced to approximately 10 cm above the patient’s eye to simulate a single-digit intra-ocular pressure.

Patients were examined on postoperative days 1, 4, 7, 14, 30, 60. If needed, laser suturelysis was performed, as described elsewhere [4].

We evaluated pre- and post-operative logMAR visual acuity. We defined complete success as IOP between 5 and 18 mmHg plus IOP reduction by at least 20% from baseline, without concomitant medications. Relative success was the same result, with glaucoma medication(s). Failure was regarded as IOP less than 5 or higher than 18 mmHg, or by the need for a subsequent glaucoma operation. 

Needling was performed when there were indications of Tenon cyst formation, with or without an associated elevation in IOP. Sub-conjunctival 5-Fluorouracil (5-FU) was injected when the bleb appeared excessively vascular or following a needling procedure. It should be noted that neither of these procedures was considered a failure of the initial surgery.

### 2.3. Statistical Analysis 

A chi-square test of independence was conducted to assess the distribution of males and females across the groups. A paired t-test was utilized to compare the mean IOP and medication use before and after surgery within each group. An independent (two-sample) t-test was employed to compare the postoperative IOP means between the trabeculectomy and phacotrabeculectomy groups. Fisher’s exact test was applied to evaluate the total and complete success rates between the two groups.

## 3. Results

We reviewed the medical records of 186 eyes of 186 patients that had undergone either trabeculectomy or phacotrabeculectomy during the three-year study period. After excluding cases with other ocular co-morbidities, 45 eyes that had undergone trabeculectomy and 35 eyes that had undergone phacotrabeculectomy were included (Table 1 and Table 2, respectively). 

**Figure 1:**
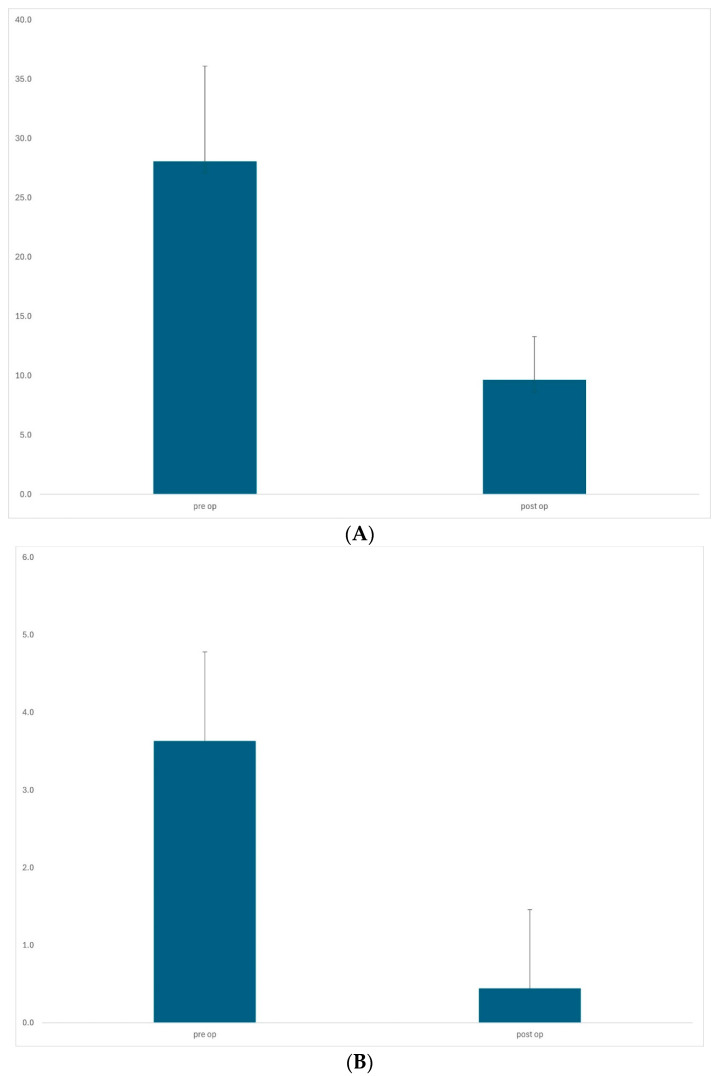
Trabeculectomy group (n = 45). The mean IOP dropped from 28.1 ± 8.0 mmHg preoperatively to 9.7 ± 3.6 mmHg (*p* < 0.0001, paired *t*-test) at the last visit, representing 66% reduction (**A**). Mean medication use fell from 3.6 ± 1.1 to 0.4 ± 1.0 (*p* < 0.0001, paired *t*-test) (**B**).

Trabeculectomy dropped the mean IOP from 28.1 ± 8.0 mmHg preoperatively to 9.7 ± 3.6 mmHg (*p* < 0.0001) at the last visit. Mean medication use fell from 3.6 ± 1.1 to 0.4 ± 1.0 (*p* < 0.0001). Mean follow-up time was 16.0 months (range 8–36), and mean IOP reduction was 66% (Figure 1). Our success rate was 88.9% (complete success 75.6%, relative success 13.3%). Mean visual acuity remained unchanged at 0.4 ± 0.3 log/MAR. The median numbers of 5FU injections and needlings were one and zero, respectively. 

**Figure 2:**
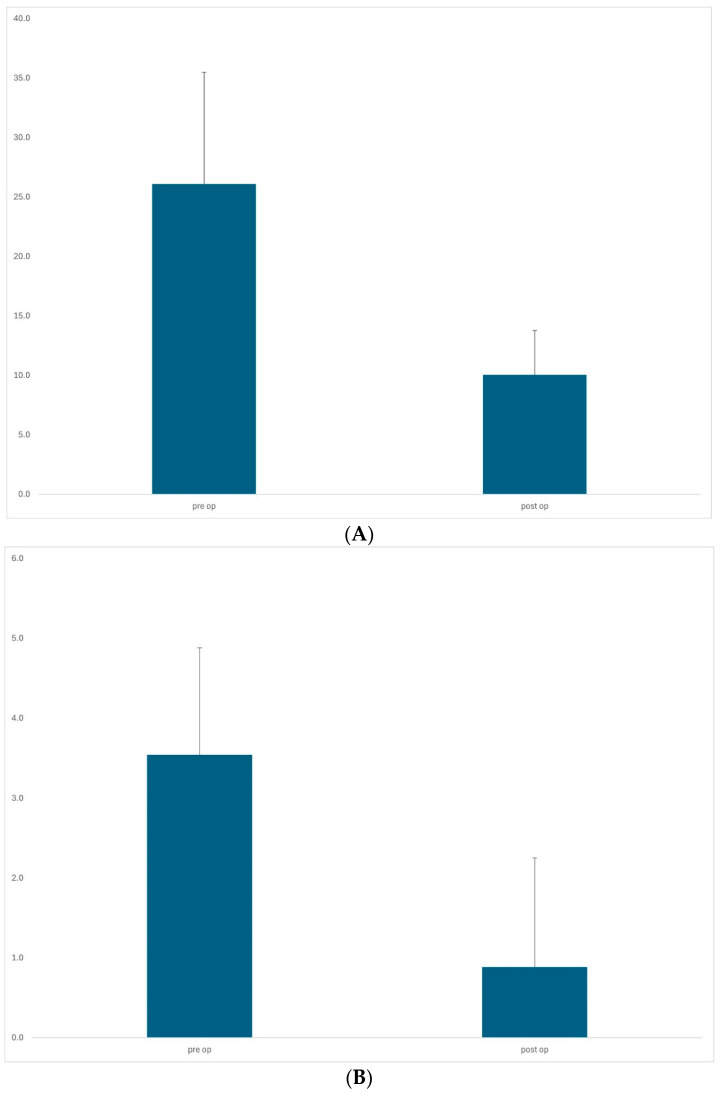
Phacotrabeculectomy group (n = 35). The mean IOP dropped from 26.1 ± 10.2 mmHg preoperatively to 10.0 ± 3.6 mmHg (*p* < 0.0001, paired *t*-test) at the last visit, representing 62% reduction (**A**). Mean medication use fell from 3.5 ± 1.3 to 0.9 ± 1.4 (*p* < 0.0001, paired *t*-test) (**B**).

In the Phacotrabeculectomy group, the mean IOP fell from 26.1 ± 9.4 mmHg to 10.0 ± 3.7 mmHg (*p* < 0.0001) post-operatively at the final review. Mean medication use fell from 3.5 ± 1.3 to 0.9 ± 1.4 (*p* < 0.0001). Mean follow-up time was 16.0 months (range 9–36) and mean IOP reduction was 62% (Figure 2). Our success rate was 91.4% (complete success 57.1%, relative success 34.3%). Preoperative baseline mean visual acuity was 0.5 ± 0.3 log/MAR, which was improved postoperatively to 0.4 ± 0.3. The median numbers of 5FU injections and needlings were two and zero, respectively. 

We encountered a few complications. Early post-operative hypotony (without concomitant choroidals or maculopathy) occurred in three cases in each group (7% of trabeculectomies and 9% of phacotrabeculectomies) but mostly resolved during follow-up. In two cases in the trabeculectomy group, the hypotony was persistent and required further surgical intervention. In five cases in the phacotrabeculectomy group, significant post-operative anterior chamber inflammation was seen on day 1 postoperatively. It was treated with sub-conjunctival steroid injections and with intracameral injection of tissue plasminogen activator (TPA). All cases resolved without sequelae.

There were no instances of shallow anterior chamber, aqueous misdirection, choroidal effusion, suprachoroidal hemorrhage, hypotony maculopathy, blebitis, or endophthalmitis.

## 4. Discussion

Glaucoma continues to be a leading cause of visual impairment and blindness worldwide, representing a significant public health challenge. The reduction of intra-ocular pressure (IOP) remains the cornerstone of glaucoma management, as it is the only modifiable risk factor proven to slow the progression of the disease and prevent further visual disability. While the majority of glaucoma patients can be effectively managed with medications and/or laser treatments, there exists a substantial subset of patients who require more invasive surgical intervention to achieve adequate IOP control.

Among the various surgical options available, trabeculectomy has long been considered by many surgeons to be the gold standard for IOP reduction in glaucoma patients. Despite the emergence of newer techniques, including minimally invasive glaucoma surgeries (MIGS) and devices such as shunts and valves, trabeculectomy remains the most reliable procedure for achieving significant and sustained lowering of IOP. Its efficacy has been well documented across numerous studies and clinical trials, solidifying its status as the preferred surgical option for patients with advanced or refractory glaucoma.

However, the widespread use of trabeculectomy is not without its challenges. The procedure, while effective, is associated with a range of potential complications that can impact both short-term and long-term outcomes. These include the risks of hypotony, bleb-related infections, and fibrosis, all of which can compromise the success of the surgery and lead to further visual deterioration. As a result, there is an ongoing and critical need to refine the trabeculectomy technique to enhance its safety profile while maintaining, or even improving, its efficacy.

The search for an ideal trabeculectomy technique—one that maximizes IOP control while minimizing the risks of complications—has been a driving force in glaucoma research and surgical practice. This pursuit is often referred to as the “holy grail” for glaucoma surgeons and patients alike, reflecting the high stakes involved in achieving a balance between safety and effectiveness. Innovations in surgical methods, such as the incorporation of anti-metabolites, improved suturing techniques, represent significant strides toward this goal. However, the quest for an optimal approach continues, as the need for a trabeculectomy technique that offers both robust IOP reduction and an improved safety profile remains a top priority in the field of glaucoma surgery.

One of the most important developments to improve the safety profile and performance of trabeculectomy was Khaw and colleagues’ “Moorfields Safer Surgery System” [2,3]. It emphasized safety measures such as an ACM, adjustable and releasable sutures for scleral flap closure, broad tissue application of MMC, and meticulous suturing to create a water-tight conjunctival closure. However, this method is time consuming and relies on post-operative suture adjustment within a limited time window to be effective. 

We present our experience with our “Flow-Adjusted Trabeculectomy”. The aim is to set the desired filtration rate during the procedure itself; most of the titration is intra-operative. We use the ACM height variations and the tension of only two fixed scleral sutures—additional lateral flap sutures only added as required. Our data suggest that this approach can achieve high rates of reproducible success with an excellent safety profile. 

With little consensus about what should be considered as “success” or “failure” in glaucoma procedures, it is challenging to compare our results with other published data. Our results are at least as successful as reported by other studies for both trabeculectomies and phacotrabeculectomies [5,6,7,8,9,10,11].

Our method has a high safety profile, which is mostly attributed to the method in which the ACM is used throughout the surgery. In the first stage of the surgery, the 50 cm height ACM stabilizes the anterior chamber. While doing the scleral punch, a 30 cm height ACM pushes the iris to the sclerostomy, guiding the surgeon to an appropriate amount of iris tissue that needs to be resected. Most importantly, in the late stage of the surgery, the low-height ACM with flow titration intra-operatively seems to simulate the “real life”, post-operative situation. Theoretically, at sea level, a 10 cm height ACM above the eye should generate approximately 8 mmHg within the eye (as one atmosphere equals 786 mmHg). While setting the scleral sutures’ tension and titrating the flow under these conditions, we minimize the post-op IOP “surprises” with greater predictability. Shallow anterior chambers were not seen post-operatively in either patient group. 

In contrast, conventional surgery relies solely on the surgeon’s estimation of suture tightness, which can lead to unpredictable outcomes, such as post-operative pressures being too high or too low. This makes the ACM particularly valuable in improving surgical precision and optimizing post-operative IOP control.

The rates of needlings in both groups was very low (median = 0). 5FU injections were given more frequently (median, 1 versus 2 in the trabeculectomy and phacotrabeculectomy groups, respectively). A pro-active approach for post-operative anti-metabolite injections may help chances of success [6].

To assess the safety of both procedures, we looked at visual acuity (VA) as an indicator. In the trabeculectomy group, the VA remained unchanged (0.4 logMAR), while the phacotrabeculectomy group showed a mild improvement in visual acuity from 0.5 logMAR pre-operatively to 0.4 logMAR post-operatively. This relatively modest improvement in visual acuity in the phacotrabeculectomy group could be explained by optic nerve damage present in advanced glaucoma patients, along with some patients undergoing cataract surgery “preemtively” to avoid a subsequent cataract extraction in the presence of a functioning drainage bleb.

In contrast to other studies [9,10], our total and complete success rates did not show a statistically significant difference between combined phacotrabeculectomy and standalone trabeculectomy procedures (91.4% vs. 88.9%, Fisher’s exact test = 1.0, and 57.1% vs. 75.6%, Fisher’s exact test = 0.0964; neither significant at *p* < 0.05). Additionally, the postoperative IOPs were not significantly different between the combined and standalone procedures (10.0 ± 3.7 mmHg and 9.7 ± 3.6 mmHg, respectively, *p* = 0.72). This outcome may be attributed to the intra-operative flow adjustments made during surgery.

Our study has several limitations. First, as a retrospective study, it relies on pre-existing medical records, which may contain incomplete, inconsistent, or inaccurate data. The study is also prone to selection bias, since the cases being reviewed are not randomly selected. Additionally, it is susceptible to recall bias, and lacks the ability to establish causality, as it can only identify associations between variables. The retrospective nature also limits control over confounding factors, which can impact the validity and the generalizability of the findings.

Second, given that our study exclusively examined patients with open-angle, pseudo-exfoliative, and pigmentary glaucoma, it remains possible, although unlikely, that the findings may not be applicable to individuals with other forms of glaucoma or those with additional ocular co-morbidities.

Third, the follow-up period ranged from 16 to 19 months, which is comparable to or exceeds the duration used in many other glaucoma studies. However, considering the chronic nature of glaucoma, our follow-up duration is relatively short. The limited follow-up duration restricts our ability to assess the long-term effectiveness of the bleb and to identify potential complications that may arise over time. Future studies with extended follow-up periods are necessary to address these concerns.

Fourth, although the sample size of our study was sufficient for statistical analysis, it remains relatively small in absolute terms. Larger-scale studies may be necessary to confirm and validate our findings regarding efficacy and safety.

## 5. Conclusions

Our “Flow-Adjusted Trabeculectomy” technique demonstrates a promising balance of safety and efficacy in the surgical management of glaucoma. The results from our clinical practice indicate that this method not only achieves significant reductions in IOP but also minimizes the risk of common complications associated with traditional trabeculectomy procedures, such as hypotony and bleb-related issues. We encourage others to independently evaluate its efficacy and safety. However, as this was a descriptive study, future controlled and prospective studies are required to validate these results.

## Figures and Tables

**Table 1:** Demographics and pre-op data of the trabeculectomy group.

Number of eyes	45
Age (years ± SD)	69.0 ± 12.4
Sex (male/female)	51.1%/48.9% Chi-square = 0.86, *p* = 0.35
Mean follow-up period (months, range)	16.0 (8–36)
BCVA (log/MAR ± SD)	0.4 ± 0.3
IOP (mmHg ± SD)	28.1 ± 8.0
IOP lowering medications (*n* ± SD)	3.6 ± 1.1

SD = standard deviation, BCVA = best corrected visually acuity, MAR = minimal angle of resolution, IOP = intra-ocular pressure.

**Table 2:** Demographics and pre-op data of the phacotrabeculectomy group.

Number of eyes	35
Age (years ± SD)	70.9 ± 7.7
Sex (male/female)	57.1%/42.9% Chi-square = 0.04, *p* = 0.83
Mean follow-up period (months, range)	19.1 (9–36)
BCVA (log/MAR ± SD)	0.4 ± 0.3
IOP (mmHg ± SD)	26.1 ± 10.2
IOP lowering medications (*n* ± SD)	3.5 ± 1.3

SD = standard deviation, BCVA = best corrected visually acuity, MAR = minimal angle of resolution, IOP = intra-ocular pressure.

## Data Availability

The datasets generated and analyzed during this study are available.
